# Acoustic Emission Analysis of Moisture Damage Mechanisms in 3D Printed Auxetic Core Sandwiches

**DOI:** 10.3390/s26031034

**Published:** 2026-02-05

**Authors:** Jean-Luc Rebiere, Abderrahim El Mahi, Zeineb Kesentini, Moez Beyaoui, Mohamed Haddar

**Affiliations:** 1 Laboratoire d’Acoustique de l’Université du Mans (LAUM), UMR 6613, Institut d’Acoustique-Graduate School (IA-GS), CNRS, Le Mans Université, 72085 Le Mans, France; abderrahim.elmahi@univ-lemans.fr (A.E.M.); zeineb.kesentini@univ-lemans.fr (Z.K.); 2 Laboratory of Mechanics Modeling and Production (LA2MP), National Engineering School of Sfax, University of Sfax, BP N° 1173-3038, Sfax 3029, Tunisia; moez.beyaoui@enis.tn (M.B.); mohamed.haddar@enis.tn (M.H.)

**Keywords:** auxetic structures, bio-based composite, 3D printing, water aging, bending test, acoustic signal classification, acoustic emission

## Abstract

This article presents an experimental investigation of the effect of water aging on the static mechanical behavior and damage mechanisms of bio-based sandwich structures with auxetic cores using acoustic emission (AE) monitoring. Both the skins and the core are manufactured by 3D printing using polylactic acid (PLA) reinforced with short flax fibers. Four auxetic core configurations, differing in the number of unit cells across the core width, are considered. The specimens are immersed in water at room temperature to characterize their absorption behavior, which follows a Fickien’s diffusion law model with different saturation levels. Static three-point bending tests are performed at various immersion times to evaluate the influence of moisture on mechanical performance. The results show a progressive degradation of mechanical properties with increasing water exposure time, with the four-cell core configuration exhibiting the highest mechanical performance. Acoustic emission (AE) monitoring is employed to analyze damage evolution as a function of hydrothermal aging. AE parameters such as amplitude, energy, and cumulative event count are used to identify and classify the different damage mechanisms. This approach highlights the effectiveness of acoustic emission for structural health monitoring and for assessing the durability of auxetic core sandwich structures subjected to moisture.

## 1. Introduction

Due to their excellent energy absorption capability, high strength-to-weight ratio, and high flexural stiffness, sandwich panels have been widely used for several decades in the aerospace, automotive, sports, and leisure industries [1]. Sandwich composites consist of a relatively thick core (foam, wood, or other materials) placed between two thin and stiff face sheets. The mechanical properties of these structures strongly depend on the constituent materials, type of fiber reinforcement, and core geometry [2]. Several studies have shown that flexural failure mechanisms and energy absorption capacity are strongly influenced by the core topology [3]. In response to increasing requirements for mechanical performance and structural reliability, the design of sandwich composites has evolved toward increasingly complex core architectures, among which conventional [4,5] and auxetic honeycomb structures are the most extensively investigated [6].

Auxetic honeycomb structures, characterized by a negative Poisson’s ratio, have attracted growing interest due to their unique mechanical properties [7], such as enhanced stiffness, improved energy absorption capability, and reduced weight [8]. The integration of auxetic cores into sandwich structures modifies not only the global mechanical response but also the nature, sequence, and intensity of damage mechanisms. This increased complexity makes the use of advanced monitoring techniques based on signal analysis and processing essential for reliable damage detection, localization, and classification [9].

Various manufacturing processes have been developed for producing these architected structures, including additive manufacturing (3D printing) [10,11,12,13]. In this context, Hamrouni et al. [14] fabricated auxetic core sandwich structures using 3D printing and analyzed the influence of different core architectures on their mechanical behavior. In parallel, environmental concerns have promoted the development of bio-based composites, particularly due to their good energy absorption capability and renewable origin [15,16,17,18,19,20,21,22]. Essassi et al. [23] investigated the static and vibrational mechanical properties of flax/PLA biocomposites, demonstrating promising mechanical and damping performances. However, a detailed understanding of the damage mechanisms associated with these materials requires measurement techniques capable of exploiting the information contained in the physical signals generated during degradation beyond conventional global mechanical indicators.

Bio-based composites remain sensitive to environmental conditions, particularly moisture. Plant fibers are mainly composed of cellulose, hemicelluloses, pectins, and lignin. Cellulose, a semi-crystalline polysaccharide, provides mechanical strength to the fibers but is also responsible for their hydrophilic behavior [24]. Numerous studies have shown that water absorption leads to a significant reduction in the stiffness and strength of plant fiber-reinforced composites [25,26,27]. This degradation is generally attributed to fiber deterioration, matrix plasticization, and damage at the fiber–matrix interface [28,29,30]. Monitoring these phenomena requires sensitive surveillance methods capable of detecting progressive, diffuse, and often early-stage damage.

In this context, acoustic emission (AE) is a particularly suitable non-destructive monitoring technique [31]. AE enables the real-time acquisition of transient signals generated by microscopic damage mechanisms. These signals can be processed using advanced signal processing techniques to extract relevant features such as amplitude, energy, duration, hit count, average frequency, and spectral content. Time–frequency analysis, combined with feature extraction and classification or clustering methods, allows for the identification and discrimination of different failure mechanisms.

Furthermore, architected metamaterial cores manufactured by 3D printing represent a new class of sandwich structures exhibiting improved performance under quasi-static and dynamic loading conditions [32,33,34]. However, few studies have combined the analysis of such structures with advanced acoustic emission signal processing, including feature extraction and event classification, to characterize damage evolution under severe environmental conditions such as humidity.

## 2. Materials and Experimental Processes

### 2.1. Materials and Manufacturing

**Figure 1:**
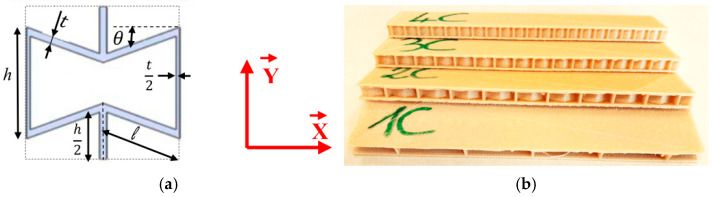
(**a**) Auxetic cell and (**b**) sandwich specimens.

The sandwich structure is made up of 100% natural constituents. The two skins and the core are manufactured with the same bio-based composite, which is polylactic acid (PLA) reinforced with short flax fiber provided by NANOVIA factory. The density of this composite is 1000 kg·m^−3^, with a short flax fiber volume fraction less than 20%. This composite is used to manufacture sandwich structures with a 3D printer, which is the MakerBot Replicator 2. Figure 1a presents the geometric characteristics of the auxetic structure employed as the sandwich composite’s core material. It is called an auxetic re-entrant honeycomb structure.

The initial lengths of the inclined and vertical cell walls are given by *l* and *h*, respectively. *θ* is the angle between the slanted cell walls and the X axis. The thickness of the auxetic core is *b*, while the thickness of the cell wall is *t*. The specimens are 25 mm in width. With the same width, we can manufacture sandwiches with different numbers of cells in the core: one S1C, two S2C, three S3C, or four S4C cells (Figure 1b). Table 1 gathers the dimensions of the auxetic unit cell used in the core of the four sandwiches.

### 2.2. Mechanical Tests

**Figure 2:**
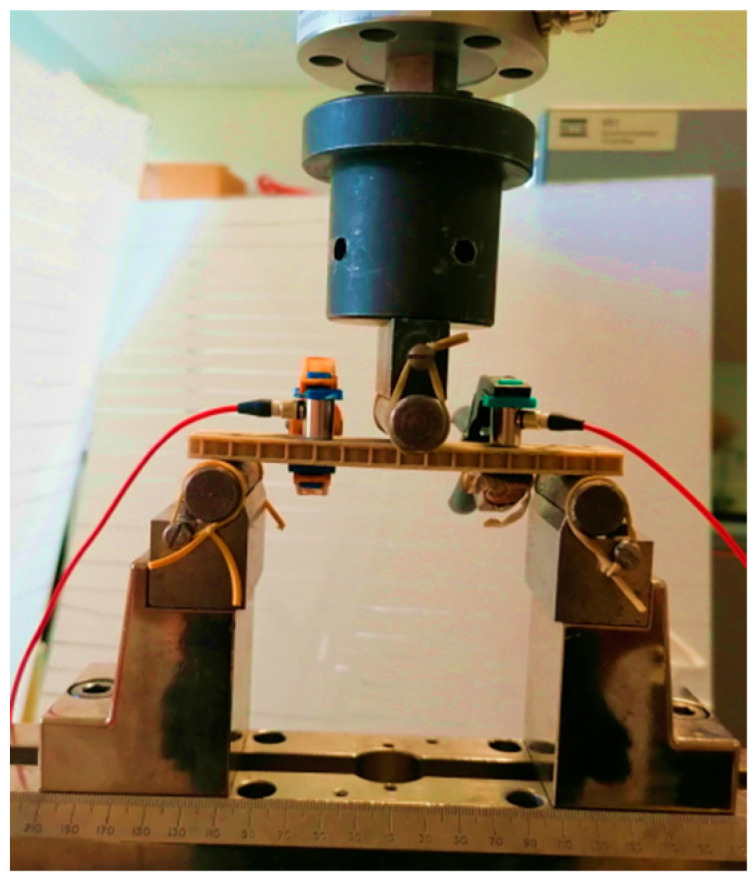
Three-point bending test.

The experiment setup used to characterize sandwich beams is three-point bending. The machine used is an INSTRON 8801 conventional hydraulic machine with 1 KN load cell (Figure 2). Static tests are carried out in accordance with the ASTM C393 standard with a 3 mm/min displacement rate. Dimensions of the sandwich specimens are 130 mm in length, 25 mm in width, and 7 mm (2 mm for the two skins and 5 mm for the core) in thickness. The span length is fixed at 110 mm. Three specimens are tested to consider experimental errors.

To determine the sandwiches’ bending and shear stiffness, three-point bending tests are also carried out within the elastic limit of sandwiches for a span length from 100 to 250 mm. Here, we tested specimens with the same width and thickness as before but with a length of 270 mm.

### 2.3. Acoustic Emission Technique

The acoustic emission technique is associated with mechanical tests in 3-point bending in order to identify and track the spread of damage mechanisms. Damages created within the sandwiches are at the origin of the appearance of the waves and thus of the acoustic signals. The acoustic emission technique consists of fixing two piezoelectric sensors on the specimen, which are linked to the computer for recording using the AEWIN software with a frequency of 5 MHz. The samples are instrumented using two special piezo-electric sensors within a bandwidth between 100 kHz and 1 MHz, branded “Mistras”. To ensure good propagation of acoustic signals, a special gel is used to ensure good coupling between the specimen and the sensors. Two clamps are used to hold the sensors relative to the specimen during the test. In order to avoid parasitic noises, a recording threshold of 38 dB has been set. This value of 38 dB, which was chosen, is the result of experimental test campaigns. The sensors are fixed midway between the loading point and the supports (Figure 2).

The recorded AE signals are filtered using two preamplifiers with a gain of 40 dB. The recording system also requires other preliminary settings for certain configuration parameters in order to define the recording time windows. They are defined as follows:

- The PDT (peak definition time) determines the rise time of the acoustic event. PDT = 50 ms.
- The HDT (Hit Definition Time) determines the end of the acoustic event. HDT = 100 ms.
- The HLT (Hit Lock-out Time) creates a dead time at the end of each acoustic event in order to exclude late reflections. HLT = 200 ms.

**Figure 3:**
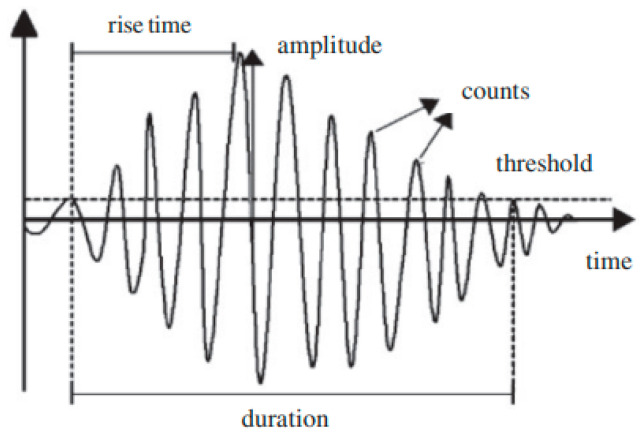
Acoustic emission waveform and parameters.

At the end of the test, the recorded acoustic data is analyzed using the NOESIS software. Based on the work carried out within the laboratory on bio-based composites, five temporal parameters are chosen for the classification. These parameters are amplitude, rise time, duration, absolute energy, and number of counts (Figure 3).

The unsupervised classification method is used by adopting the k-means algorithm. This method consists of separating the data into an optimal number of classes. It is an iterative approach to data partitioning that minimizes within-group variance, or the sum of the squares of the distances between all elements of a class and its center. All data have been normalized to compare the magnitudes of descriptors (parameters) that do not have the same unit and to prevent one descriptor from becoming more significant than the others. Then, the k-means algorithm can be used. Initially, a random segmentation is applied with a well-chosen Euclidean distance [35,36]. Then, the algorithm is applied several times by sequences of 1000 iterations by varying the classes from 2 to 5. At each step, the Davies and Bouldin coefficient [37] is calculated by Equation (1):

(1) $$R_{ij}\left(D\&B\right)=\frac{1}{K}\sum_{i=1}^{n}{max}_{i}\left(\frac{d_{i}+d_{j}}{d_{ij}}\right)$$

where *K* is the number of classes, *d*_i_ and *d*_j_ are, respectively, the average distances in classes *i* and *j*, and *d*_ij_ is the average distance between classes *i* and *j*. The lowest value of this coefficient is the optimal number of classes.

### 2.4. Aging Conditions

The purpose of this research is to examine water aging effects on the static behavior of sandwich structures and on the propagation of damage mechanisms. Five specimens of each sandwich configuration are immersed in tap water at room temperature. They are removed to be weighed and calculate the water uptake following Equation (2). This step is repeated periodically until saturation [28,38]:

(2) $$M_{t}=\frac{W_{t}-W_{0}}{W_{0}}\times 100(\%)$$

where *W*_0_ is the mass of the non-immersed sample at *t* = 0, and *W*_t_ is the mass of the samples after a given immersion time t.

Mechanical tests and the recording of damage mechanisms are carried out on specimens for different immersion times.

## 3. Results and Discussion

### 3.1. Water Absorption of Sandwich Composites

To study the effect of water absorption on sandwich structures, it is necessary to know the absorption behavior and the percentage of water uptake at saturation. For that reason, the percentage of absorption obtained by Equation (2) is plotted as a function of the immersion time. Different models can describe the absorption behavior. In this study, Fick’s model is adopted based on results generated from the following equations [39,40]:

(3) $$\frac{M_{t}}{M_{m}}=1-\exp[-7.3\times (\frac{D_{i}\cdot t}{h^{2}}{)}^{0.75}]$$

where *h* is the thickness of the solid subjected to diffusion, $D_{i}$ is the diffusion coefficient, *t* is the diffusion time, *M*_t_ is the water content at time *t*, and *M*_m_ is the maximum mass of water at saturation.

(4) $$D_{i}=\pi \times (\frac{k}{4M_{m}}{)}^{2}$$

where *k* is the slope of the linear part of the curve *M*_t_ = *f* (√*t*/*h*).

**Figure 4:**
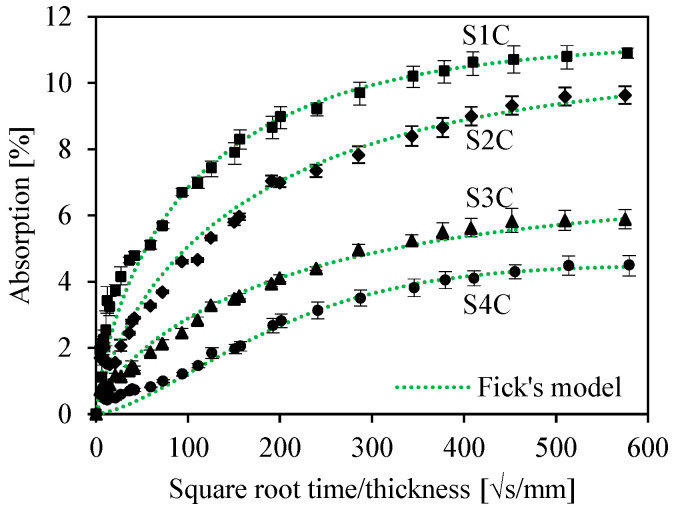
Evolution of water uptake of sandwiches.

Figure 4 depicts the water uptake curves for the four sandwiches as a function of the square root of immersion time. The absorption percentage increases according to the immersion time until saturation. It should also be noted that the absorption curves increase linearly as a function of the square root of the time at the beginning and then slow down until saturation. This behavior corresponds well to the behavior of the Fick model, which is represented with the green curves. The water absorption in the sandwiches is the combination of water absorbed by the skin and by the core constituting the sandwich. In our case, the skins and the core are made of the same composite material. The difference in absorption and saturation behavior is due to the cell numbers in the core. It is noted that the sandwich with a single cell presents a sudden rise in the rate of absorption at the beginning. This increase can be explained by the cavities created within the sandwich. Water is trapped in the spaces of the sandwich’s core.

Chemically, three forms of water are obtained. There is absorbed (free) water, which has no chemical interaction with the material; bounded water, where the molecules enter the molecular structure; and adsorbed water (on the surfaces). For four-cell sandwiches, the diffusive behavior is close to that of the skin [28]. By increasing the number of cells, the structure of the sandwich becomes more and more compact, which prevents the total wetting of the sandwich core. It should be noted that, for sandwich structures, the water absorption rate decreases when the density increases. Adverse effects of aging can occur, which influences the life of PLA composites reinforced with plant fibers.

### 3.2. Effect of Water Absorption on the Static Behavior

**Figure 5:**
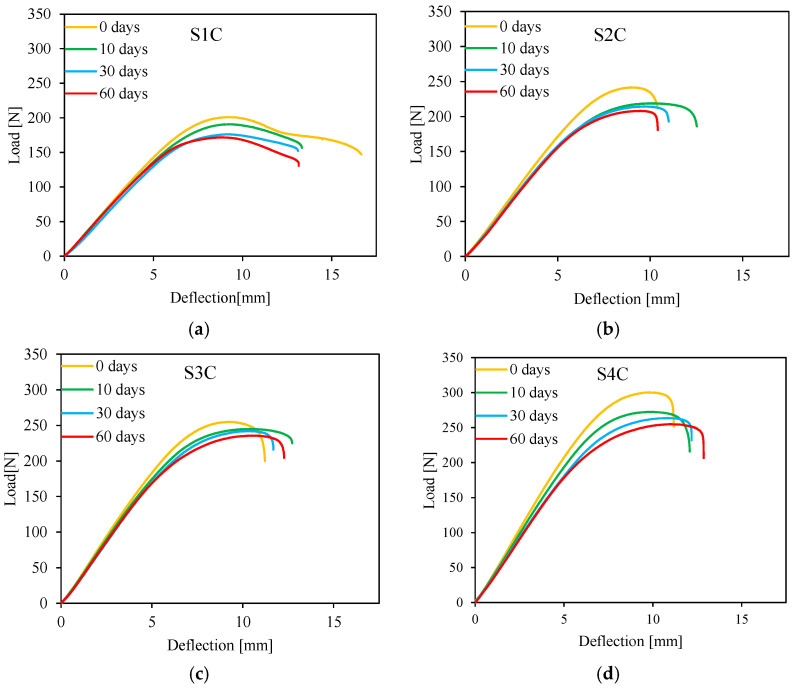
Load/deflection curves for different immersion times: (**a**) 1 cell, (**b**) 2 cells, (**c**) 3 cells, and (**d**) 4 cells.

The bending behavior of sandwiches with different numbers of cells for different immersion times is studied. Three-point bending is carried out for each configuration with a 110 mm span length. The experimental results of four immersion times are shown in Figure 5. The four configurations exhibit the same behavior for the different immersion times. Each curve has three parts: linear elastic part, nonlinear plastic part, and failure part. The four-core cell sandwich presents a high load value. Moreover, the linear elastic zone appears larger than the one-core cell sandwich. It can also be observed that the configuration with a small number of cells exhibits a dispersion of the failure characteristics.

Comparing the four immersion times chosen and for the four sandwiches, we observe that the curves for the different aging times are similar, with a higher maximum value for the non-aged sandwich. Peak strength and stiffness decrease as the immersion time increases.

Three-point bending tests are performed on the four sandwich structures for seven immersion times, and the failure characteristics are evaluated. The bending stress *σ* of the sandwich beams is given by Equation (5) [ASTM C393/C393M]:

(5) $$\sigma =\frac{Pd}{2e_{p}\left(h+e_{a}\right)b}$$

where *d* is the span length and *e_p_* and *e_a_* are the thicknesses of the two skins and the core, respectively. *b* and *h* present the width and the thickness of the specimen, respectively.

Moreover, the core shear stress τ is calculated by Equation (6) [ASTM C393/C393M]:

(6) $$\tau =\frac{P}{\left(h+e_{a}\right)b}$$

**Figure 6:**
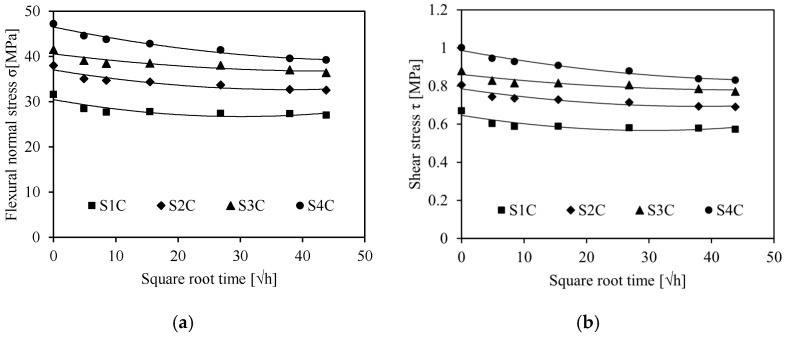
(**a**) Flexural normal stress, (**b**) shear stress of the sandwiches as a function of different immersion times.

Figure 6 shows the experimental results obtained for the four sandwiches for the different immersion times. These results show that, as the immersion time increases from 0 days to saturation, the flexural normal stress and the shear stress are affected.

Non-aged sandwiches exhibit optimal flexural and shear stresses, increasing with the number of cells in the sandwich core structure. At saturation level, 15% is the loss of the flexural and shear stresses for all sandwiches. Hence, we can conclude that water aging considerable degrades the mechanical characteristics of flax/PLA sandwiches with an auxetic core. This decline with aging can be caused by the degradation of the constituents due to the penetration of water into the macromolecular chains of the composite, which causes damage.

In order to determine the elastic characteristics of the sandwich structures, the four configurations’ structures are tested with three-point bending in their elastic domains by varying the span length from 100 to 250 mm and for the four chosen immersion times. From these tests, the shear modulus *G*_a_ and the Young’s modulus of the skins *E*_p_ are deduced. A sandwich composite’s deflection is the combination of bending and shear components. The tensile and compressive moduli of the skins determine the amount of bending deflection. Shear deflection is determined by the sandwich core’s shear modulus. For a sandwich composite with an auxetic core supported with a central load, the equation describing the deflection *W* with the applied load *P* in three-point bending tests is given by [41]:

(7) $$\frac{W}{\mathrm{Pd}}=\frac{d^{2}}{48D}+\frac{1}{4N}$$

where *d* is the span length, *D* is the bending stiffness, and *N* is the shear stiffness.

**Figure 7:**
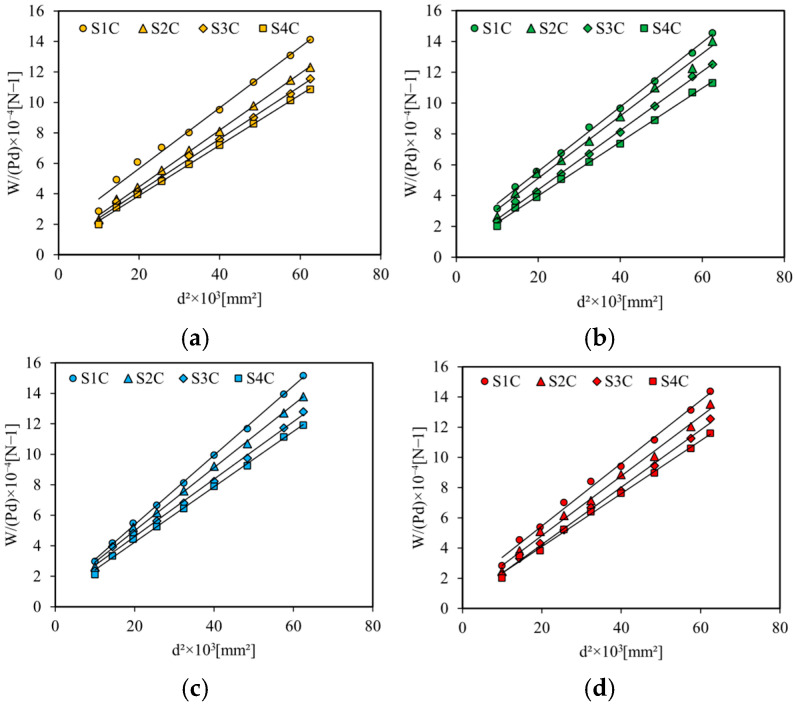
Evolution of P/(Wd) ratio as a function of d^2^ for the four configurations at (**a**) 0 days, (**b**) 10 days, (**c**) 30 days, and (**d**) 60 days.

Thus, by varying the distance *d*, the curve of *W*/(*Pd*) as a function of *d*^2^ makes it possible to obtain a straight line (Figure 7). From the slope and the interception point of this curve, we can determine *D* and *N*, respectively.

In addition, the bending and the shear stiffness can be calculated according to ASTM C393/C393M [41] with Equations (8) and (9):

(8) $$D=\frac{E_{p}be_{p}{\left(e_{p}+e_{a}\right)}^{2}}{2}$$

(9) $$N=b\left(e_{p}+e_{a}\right)G_{a}$$

*e_p_* and *e_a_* are the thicknesses of the two skins and the core, respectively, and *b* presents the width of the specimen.

**Figure 8:**
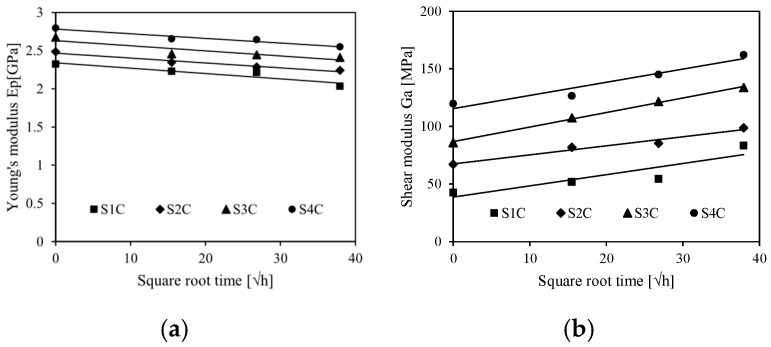
Properties of the constituents of the sandwiches for different immersion times: (**a**) Young’s modulus of the skins and (**b**) shear modulus of the core.

Thus, the Young’s modulus of the skins *E*_p_ and the shear modulus of the cores *G*_a_ are determined and presented as a function of the number of cells in Figure 8.

On one hand, we note that the Young’s modulus of the skins increases with the number of cells, which is obvious, and decreases with the immersion time. The decrease in Young’s modulus is attributed to the penetration of water molecules into the macromolecular chains of the flax/PLA biocomposite structure. On the other hand, the shear modulus increases with the immersion time. This is due to the crystallization of the chains of the composite structure following the penetration of water. This behavior has been revealed in several studies. For example, Osman et al. [42] worked on composites reinforced with bio-based fibers. They showed that the bending properties decrease with immersion time. Prabhakaran et al. [43] are interested in the effect of water absorption on the flexural properties of bio-sourced sandwich composites. The bending tests have drawn the conclusion that the mechanical properties of the sandwiches increase with the cells’ number of cores and decrease as function of the immersion time.

## 4. Damage Mechanisms of the Sandwiches

**Figure 9:**
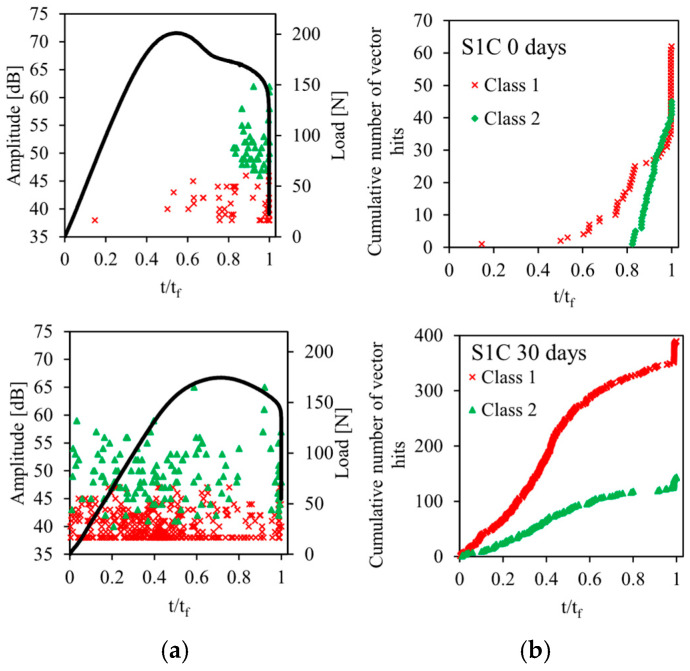
Load vs. time and AE data points for different immersion times of sandwich with 1 cell: (**a**) Events Amplitude and stress versus time and (**b**) chronology of appearance of different classes.

**Figure 10:**
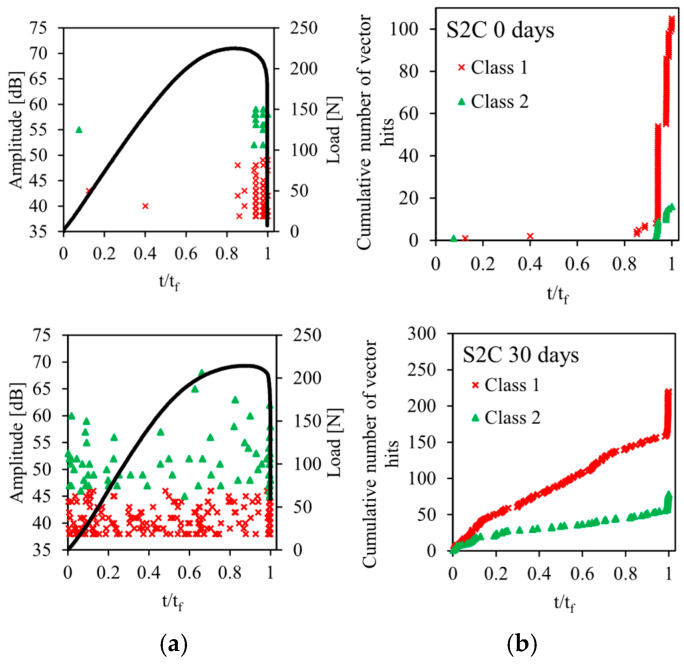
Load vs. time and AE data points for different immersion times of sandwich with 2 cells: (**a**) Events Amplitude and stress versus time and (**b**) chronology of appearance of different classes.

**Figure 11:**
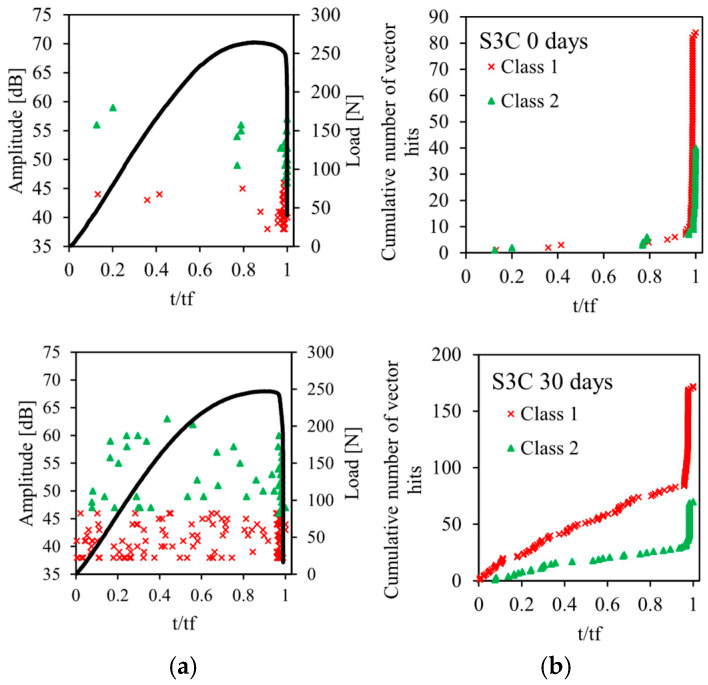
Load vs. time and AE data points for different immersion times of sandwich with 3 cells: (**a**) Events Amplitude and stress versus time and (**b**) chronology of appearance of different classes.

**Figure 12:**
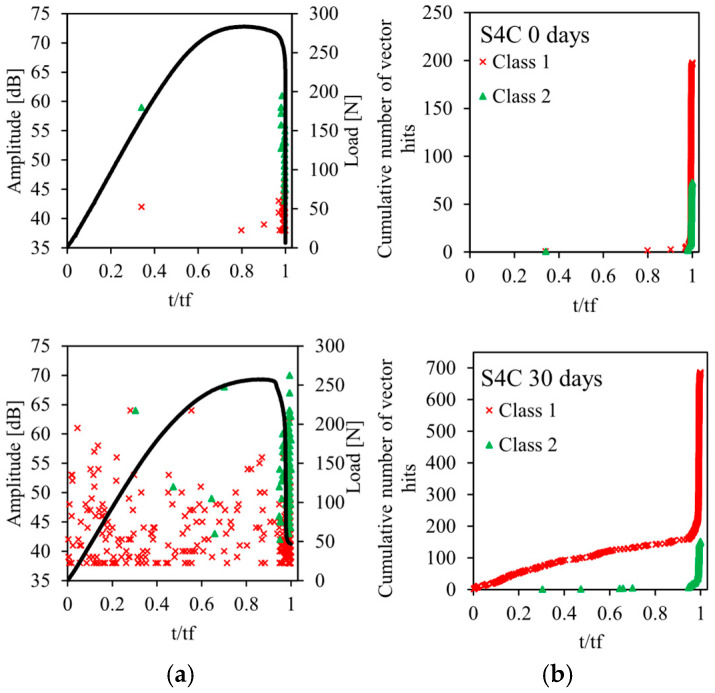
Load vs. time and AE data points for different immersion times of sandwich with 4 cells: (**a**) Events Amplitude and stress versus time and (**b**) chronology of appearance of different classes.

**Figure 13:**
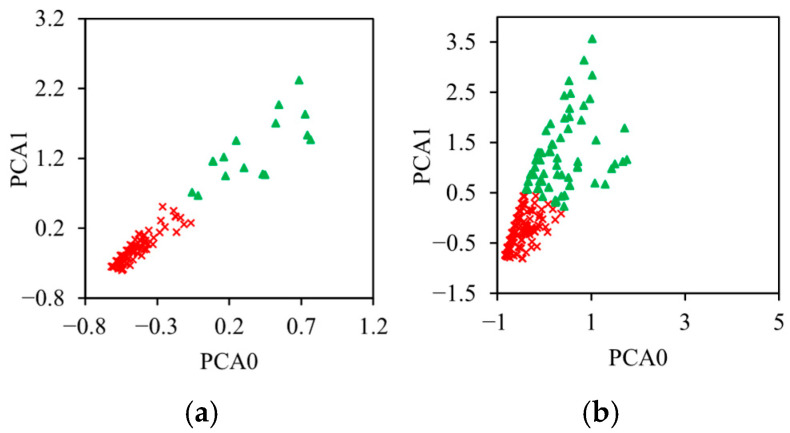
Principal component analysis of sandwich composites with 2 cells for two immersion times: (**a**) 0 days and (**b**) 30 days.

The acoustic emission method described in Section 2.3 is used for the recording and for the classification of acoustic emission signals emitted during static bending tests on sandwiches. The results of this analysis are shown in Figure 9, Figure 10, Figure 11, Figure 12 and Figure 13 for the four sandwiches and for different aging times (non-aged and aged 10, 30, and 60 days). Figure 9a, Figure 10a, Figure 11a and Figure 12a represent the amplitude distribution associated with the evolution of the load as a function of time. These results show that the acoustic activity of the four non-aged sandwiches only begins at the end of the test, unlike the aged sandwiches. For the latter, acoustic events appear from the beginning of the test, which is caused by the degradation of the material. Visually, a crack appears in the bottom skin at the start of the bending test. Following that, the break spreads to the auxetic core. The bottom skin and the core structure began to debond toward the end of the test. In addition, for the four sandwiches, there are two acoustic classes, with amplitudes varying from 38 dB to 75 dB. Therefore, the damage of non-aged and aged sandwiches is mainly caused by matrix cracking and fiber/matrix debonding. The first damage mechanism is characterized by low amplitudes between 38 dB and 50 dB. The second damage mechanism, which evolves until the total rupture of the specimens, is characterized with amplitudes between 45 dB and 75 dB.

The evolution of the cumulative number of vector hits of the different acoustic classes detected as a function of time are represented in Figure 9b, Figure 10b, Figure 11b and Figure 12b. The cumulative number of vector hits increases with the number of cells in the core of the sandwich. At the end of the test, and for the non-aged sandwich with a single cell in the core, this number is around 105, whereas it is 270 for a non-aged sandwich with four cells. Finally, these results also highlight the effect of humidity on damage mechanisms. Indeed, for non-aged sandwiches, the acoustic events detected appear gradually during the test, then accelerate towards the end of the test. For aged sandwiches, the events start from the beginning of the test. This is due to the damage of the sandwich’s constituents caused by the absorption of water. This absorption phenomenon leads to early damage, in particular, at the fiber/matrix interface. The accumulation of events at the beginning of the test indicates initial damage. This finding justifies the significant loss of mechanical properties—essentially, the Young’s modulus.

Principal component analysis (PCA) in Figure 13 is also carried out to visualize the results in a two-dimensional subspace. Figure 13 shows an example of the principal component analysis of the sandwich with two cells in the core for a non-immersed specimen and a specimen immersed for 30 days.

That makes it possible to highlight the separation between the two classes from the classification [44]. The representation shows a clearer partition of the events in the PCA1 versus PCA0 plots. This separation of the classes is observed for the different sandwiches and for different immersion times, which validates the k-means algorithm.

## 5. Conclusions

In this article, acoustic emission (AE) technology is used to identify and monitor the damage mechanisms created by the effect of water aging on the static bending properties (three-point bending) of bio-based sandwich composites with an auxetic core. The bio-based material, PLA reinforced with short flax fibers (less than 20%), is used to manufacture the sandwich panels. Numerous experiments are carried out on four core densities.

The main results are as follows:

- Two types of damage mechanisms are detected by acoustic emission: matrix cracking and fiber/matrix delamination.
- Water aging accelerates the onset of damage and its propagation in the bio-based composite material.
- Reduction in mechanical properties.
- The mechanical properties of the four-cell sandwich panels are superior to those of the others.

Reinforcing the analysis of acoustic emissions with microscopic observations could be important for this study. In future work, the application of fatigue tests and numerical simulation with the impact of water aging would be useful to evaluate damage mechanisms and determine the service life of aged sandwich panels.

## Figures and Tables

**Table 1:** Sizes of auxetic cores.

Cells in Width	*l* (mm)	*h* (mm)	*θ* (degree)	*t* (mm)	*b* (mm)
S1C: 1 cell	13.3	17.04	−20	0.6	5
S2C: 2 cells	6.65	8.52	−20	0.6	5
S3C: 3 cells	4.43	5.68	−20	0.6	5
S4C: 4 cells	3.32	4.26	−20	0.6	5

## Data Availability

The original contributions presented in this study are included in the article. Further inquiries can be directed to the corresponding author.
